# Trimeric Architecture Ensures the Stability and Biological Activity of the Calf Purine Nucleoside Phosphorylase: In Silico and In Vitro Studies of Monomeric and Trimeric Forms of the Enzyme

**DOI:** 10.3390/ijms24032157

**Published:** 2023-01-21

**Authors:** Alicja Dyzma, Beata Wielgus-Kutrowska, Agnieszka Girstun, Zoe Jelić Matošević, Krzysztof Staroń, Branimir Bertoša, Joanna Trylska, Agnieszka Bzowska

**Affiliations:** 1 Division of Biophysics, Institute of Experimental Physics, Faculty of Physics, University of Warsaw, Pasteura 5, 02-093 Warsaw, Poland; 2 Department of Molecular Biology, Institute of Biochemistry, Faculty of Biology University of Warsaw, Miecznikowa 1, 02-096 Warsaw, Poland; 3 Department of Chemistry, Faculty of Science, University of Zagreb, Horvatovac 102a, HR-10000 Zagreb, Croatia; 4 Centre of New Technologies, University of Warsaw, Banacha 2c, 02-097 Warsaw, Poland

**Keywords:** purine nucleoside phosphorylase, homooligomeric proteins, obligate (obligatory) oligomer, subunit–subunit interface, rearrangement of the active site, tertiary/quaternary structure, in silico/in vivo/in vitro

## Abstract

Mammalian purine nucleoside phosphorylase (PNP) is biologically active as a homotrimer, in which each monomer catalyzes a reaction independently of the others. To answer the question of why the native PNP forms a trimeric structure, we constructed, in silico and in vitro, the monomeric form of the enzyme. Molecular dynamics simulations showed different geometries of the active site in the non-mutated trimeric and monomeric PNP forms, which suggested that the active site in the isolated monomer could be non-functional. To confirm this hypothesis, six amino acids located at the interface of the subunits were selected and mutated to alanines to disrupt the trimer and obtain a monomer (6Ala PNP). The effects of these mutations on the enzyme structure, stability, conformational dynamics, and activity were examined. The solution experiments confirmed that the 6Ala PNP mutant occurs mainly as a monomer, with a secondary structure almost identical to the wild type, WT PNP, and importantly, it shows no enzymatic activity. Simulations confirmed that, although the secondary structure of the 6Ala monomer is similar to the WT PNP, the positions of the amino acids building the 6Ala PNP active site significantly differ. These data suggest that a trimeric structure is necessary to stabilize the geometry of the active site of this enzyme.

## 1. Introduction

In some oligomeric proteins, subunits must be assembled before the protein becomes functional. For example, this is the case for enzymes that have a catalytic site located at the interface between neighbouring subunits, the so-called shared active site. One such example is an aspartate transcarbamoylase (ATCase), which forms a dodecameric complex, composed of two catalytic trimers and three regulatory dimers. Catalytic subunits build the active site at the interface between two trimers [1,2]. By contrast, in some homooligomeric proteins, the binding of ligand or catalytic functions are performed only by a single subunit, whereas the rest of the complex serves as an allosteric regulatory unit. This phenomenon is known as part-of-the-sites binding or reactivity [3]. One example of such is the homodimeric cyclooxygenase-1 (COX-1). Crystal structures of COX-1 monomers and homodimers, obtained in the presence and absence of ligands and inhibitors, revealed the details of the half-of-the-sites COX catalytic mechanism [4].

However, in many cases, it is still unclear why the proteins adopt a homooligomeric structure. One example is the mammalian purine nucleoside phosphorylase (PNP). According to [5,6], the trimeric PNP exhibits third-the-sites reactivity and one-third-of-the-sites binding in the transition state. However, the subsequent work proved no negative cooperativity between subunits, neither in the ground, nor in the transition, state [7]. In addition, the trimeric PNP showed the stoichiometry of three ligand molecules, including the transition state analogue inhibitor, immucillin, bound per enzyme trimer [7]. This discrepancy was explained in our previous work [3,8]. In light of this evidence, it is clear that oligomerization of the trimeric PNP cannot be explained by a strong negative cooperativity (one-third-of-the-sites binding) of the subunits forming this enzyme. Moreover, the oligomeric subunit association must arise from other reasons, for example, it may be due to the specific structural properties of the PNP active site or subunits. However, these are only hypotheses, as detailed reasons for adopting homo-oligomeric architecture by trimeric PNPs have not been investigated so far.

Nevertheless, these speculations are partially supported by molecular dynamics simulations and experimental studies of the enzyme from the same family, namely *Escherichia coli* PNP, which in vivo exists as a complex of three dimers, e.g., [9,10,11]. We have shown and explained [8] that the mutated form of this enzyme, which does not form the hexameric assembly, but is just a dimer, practically does not have catalytic activity. Not only a dynamic mixture of monomeric and dimeric forms was observed in this case, but even the dimer was unable to adopt the active site geometry required for catalysis. In agreement, only the residual catalytic activity was observed for this mutant, which was six orders of magnitude smaller than that exhibited by the naturally occurring hexamer. This shows that, although the catalysis is performed by the dimer, the hexameric architecture of *E. coli* purine nucleoside phosphorylase is mandatory for the biological activity of this enzyme, as it provides a stable structure of the active site in dimers forming the hexamer, which is necessary for the catalysis [8].

Purine nucleoside phosphorylase (PNP, EC 2.4.2.1) is an enzyme involved in the metabolism of purines, purine nucleosides, and nucleotides, catalyzing reversible phosphorolytic cleavage of the glycosidic bond of purine nucleosides: (deoxy)purine nucleoside + orthophosphate ↔ purine base + (2’-deoxy)ribose-1-phosphate. Two main PNP subfamilies are known, homohexameric and homotrimeric, which differ in specificity, as hexamers typically accept various purines and purine nucleosides as substrates, while trimers’ activity is restricted to 6-oxo-purine ribo- and 2′-deoxyribonucleosides and its respective purine bases. In the latter case, hydrogen bonds between the catalytic Glu201 and Asn243 with the N1 and O6 positions of the purine base, respectively, are crucial for catalysis [12].

**Figure 1:**
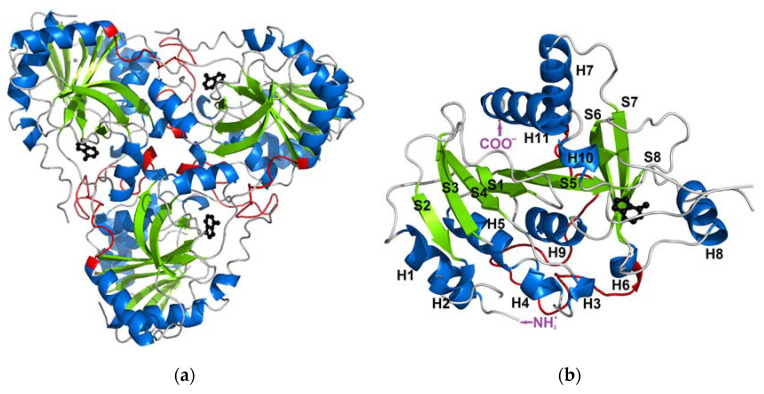
The structure of the trimeric, biologically active, calf purine nucleoside phosphorylase in the complex with one of its substrates, hypoxanthine (PDB ID: 1VFN [13]). Secondary structure elements: alpha helices and beta sheets are colored blue and green, respectively (**a**) The overall structure of the trimeric enzyme. Direct contacts between the monomers forming the trimer are mainly from the long loop (shown in red) between residues 141 and 168 of one monomer, which is the longest loop belting the monomer from one side. (**b**) The structure of one monomer showing the notation of the secondary structure elements.

When it comes to the structure, mammalian PNPs form a homo-trimeric assembly (Figure 1a) with a molecular mass of each subunit of about 30 kDa (e.g., 31.7 kDa for calf PNP) [13,14]. Each monomer is built of about 300 (289 for calf PNP; below also data for this particular PNP are given) amino acid folded to form a central β-sheet flanked by several α-helices (Figure 1b). Over 40 amino acids of each monomer contribute to the monomer–monomer interface and mainly involve the long loop between residues 141 and 168 of one monomer and two regions, namely amino acids 87–91 and 196–205 of the neighbouring monomer. Subunits are held together mostly by 15 hydrogen bonds along the interface (calculated by the PISA program [15]). The active site is located close to the border of two monomers within a trimer, but all amino acids forming the active site belong to one subunit. The only exception is Phe159, which seems to participate in the creation of the active site of the neighbouring subunit, but does not interact directly with the substrate. However, the mutation of Phe159 into alanine in the human PNP does not disrupt the monomer–monomer interface, and the enzyme exists as a trimer. It is worth noting that this mutation does not significantly change the PNP enzymatic activity [16]. Moreover, since the active site is present in each subunit, and each monomer functions independently, with no allosteric cross-talk [7], one can further speculate that, in this enzyme, the monomer is a minimal catalytic unit. Some time ago, a monomeric active form of PNP from *Thermus thermophilus* was reported [17]. This possibility can be further supported by the occurrences of other homomeric enzymes, e.g., alkaline phosphatase and triosephosphate isomerase, which are not obligate (obligatory) oligomers and are broken down into subunits to maintain an activity similar to that of the wild-type oligomeric form [18,19].

In our recent studies of the trimeric calf PNP natural aging process, we have shown that the loss of enzymatic activity of the wild-type enzyme correlates with the degradation of its trimeric structure, subsequently leading to aggregation. However, we have not observed individual, stable subunits as intermediates of the obsolescence process [20].

The question, thus, arises regarding why the calf PNP enzyme is a homotrimer. The literature data suggest that studying stable monomers of this enzyme should contribute to answering this question. Therefore, the main scope of our research was to obtain, in silico and in vitro, a mutated calf PNP with disrupted interactions between subunits. As a result, such protein should exist as a monomer, instead of a trimer, which should allow for comparing its properties with that of the wild-type PNP. With this approach, we aim to answer the question of whether the monomer of this enzyme can perform the catalysis or whether the trimeric form is obligatory for the stability, solubility, and biological activity of this protein. 

## 2. Results

### 2.1. In Silico Studies of Trimeric and Monomeric PNP Forms (WT3 PNP and WT1 PNP)

To predict whether the naturally existing calf PNP homotrimer could remain stable and active in the monomeric form, 150 ns long molecular dynamics (MD) simulations were performed for the trimeric biologically active wild-type enzyme (WT3 PNP) and its monomeric form, WT1 PNP, extracted from the trimeric X-ray structure (PDB code: 1PBN) [21]. Each MD simulation was carried out at least twice (as replicas with different starting velocities), and both calculated trajectories were analyzed to track the possible differences between the two enzyme forms.

#### 2.1.1. WT1 PNP and WT3 PNP Structural and Dynamical Properties

**Figure 2:**
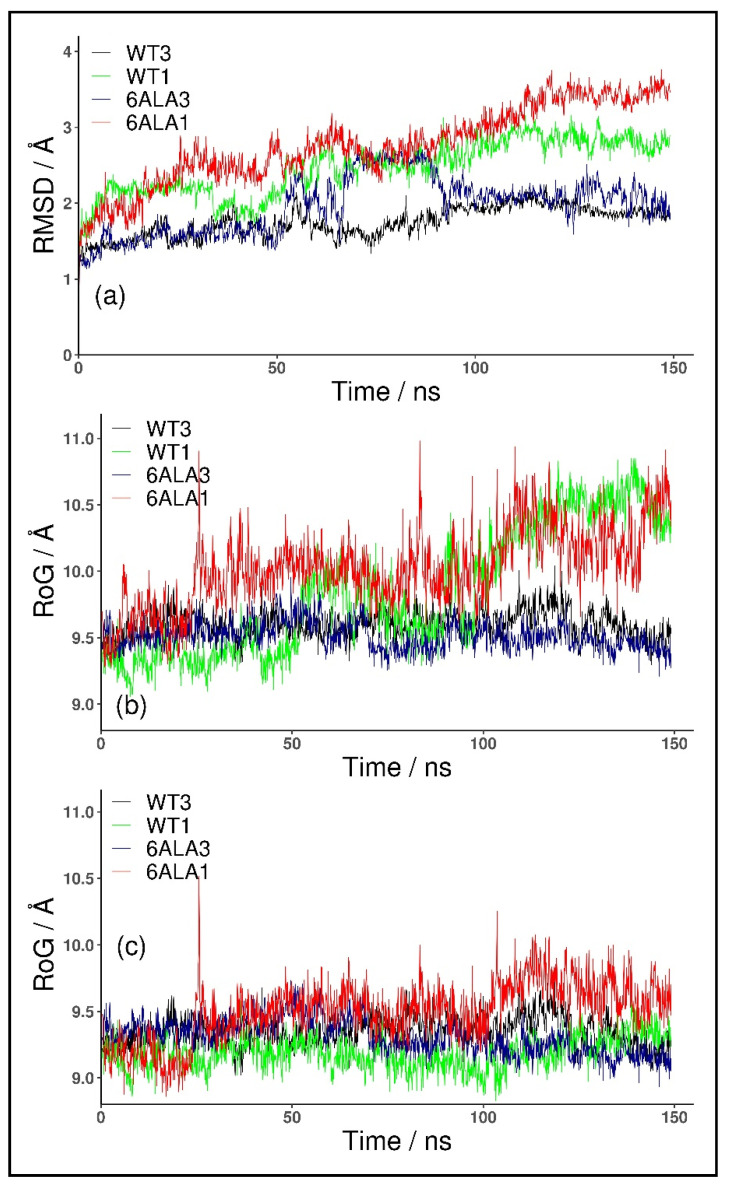
RMSD and RoG for the trajectory obtained for the trimeric biologically active enzyme, WT3 PNP (black); the monomeric form, WT1 PNP (green), derived in silico from the X-ray structure of the trimer; 6Ala3 trimeric (blue) and 6Ala1 monomeric (red) forms of the 6Ala mutant of PNP (obtained in silico). RMSD of C_α_ all atoms, calculated with respect to the starting structure (**a**) and RoG of the active site amino acids with (**b**) and without (**c**) the His64 residue included in the RoG calculation are shown.

For the monomeric form, WT1 PNP, the conformational changes of the protein were observed in both of the simulated replicates. These occurred because the parts forming the monomer–monomer interface in the trimer are mostly hydrophobic, and in the monomeric form, they became exposed to solvent. The changes were quantified with the root mean square deviation (RMSD) of the backbone Cα carbon atoms. As depicted in Figure 2a, the RMSD values plotted as a function of the simulation time, for both structures, WT3 PNP (black line) and WT1 PNP (green), stabilized with the averages and standard deviations over the last 50 ns of the simulation, equal to 1.94 ± 0.1 Å and 2.84 ± 0.1 Å, respectively, hence the difference is significant.

Moreover, we observed that the monomeric WT1 PNP tends to lose the original shape of the active site. To quantify this, we analyzed the radius of gyration (RoG) of the residues forming the active site. In the simulation of the WT1 PNP, the loop containing His64 moves away from the active site, causing a large increase in its radius of gyration. Thus, for comparison, we calculated the radius of gyration for the PNP active site with and without the His64 residue. For the WT1 PNP, with the His64 included, the average RoG of the active site in the last 50 ns of the simulation equals 10.43 ± 0.2 Å and is about 0.8 Å larger than for the trimeric WT3 PNP (9.61 ± 0.1 Å) (Figure 2b). However, if the RoG is calculated without the His64 residue, the RoG of the active site for the two forms of PNP are fairly similar (9.24 ± 0.13 Å for WT1 PNP and 9.35 ± 0.14 for WT3 PNP in the last 50 ns of the simulation) (Figure 2c).

**Figure 3:**
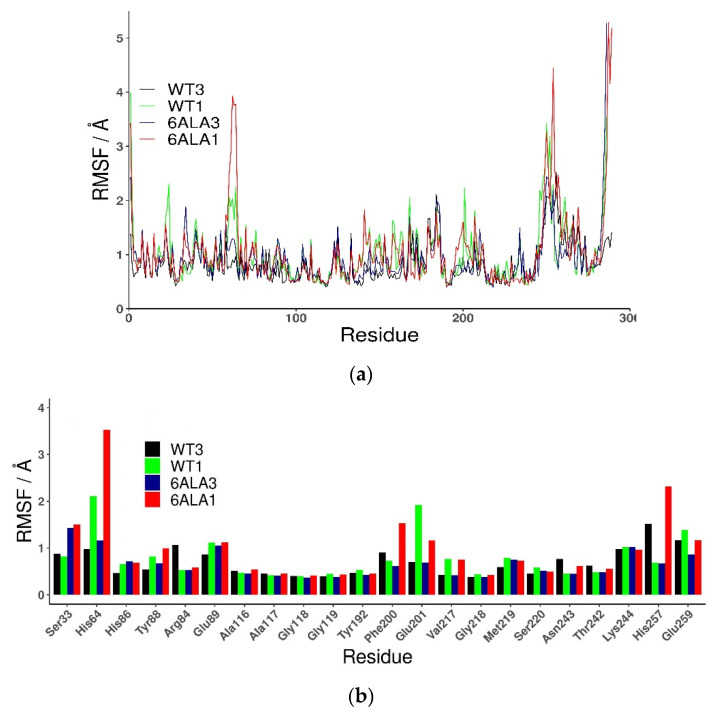
Root mean square fluctuations (RMSF) calculated for the last 50 ns of the simulation of all (**a**) and active site amino acids (**b**), observed in MD simulations for various PNP forms: trimeric WT3 PNP (black); monomeric WT1 PNP (green), trimeric 6Ala3 mutant (blue), and monomeric 6Ala1 mutant (red).

**Figure 4:**
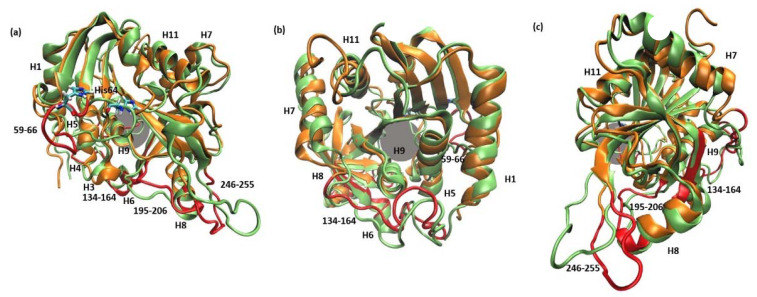
Superposition of two MD trajectory snapshots of the WT1 PNP (orange) and one subunit of WT3 PNP (green). The gray area marks the approximate location of the active site. The largest displacements between both variants are marked red and involve: the loop 59–66, on which His64 is placed, best visible on panel (**a**) (His64 side chain in both enzyme variants is shown, colored by the atom types); a large loop belting the monomer from one side and spanning residues 134–164, best visible on panel (**b**); and the helix 195–206 and the loop 246–255, which are placed adjacently, as well as on the other side of the inter-dimer interface from the loop 134–164, see panels (**a**,**c**). The exemplary snapshots are after 150 ns of MD simulation.

The root mean square fluctuations (RMSF) show (Figure 3) that the largest differences between the WT3 PNP and WT1 PNP fluctuations occur in the regions 59–66 (the loop containing His64), 134–164 (a large loop belting the monomer from one side and spanning the inter-dimer and inter-trimer interface, see Figure 1), and the helix 195–206 and loop 246–255, which are placed adjacently, as well as on the other side of the inter-dimer interface from the previously mentioned loop (Figure 4). The regions of the protein that are solvent-exposed in the trimeric form have similar RMSF values for the WT1 PNP and WT3 PNP in the conducted simulations. The active site residues for which the RMSF is significantly larger for the WT1 PNP are His64, His86, Tyr88, Glu89, Glu201, Val217, and Glu259 (Figure 3b).

**Figure 5:**
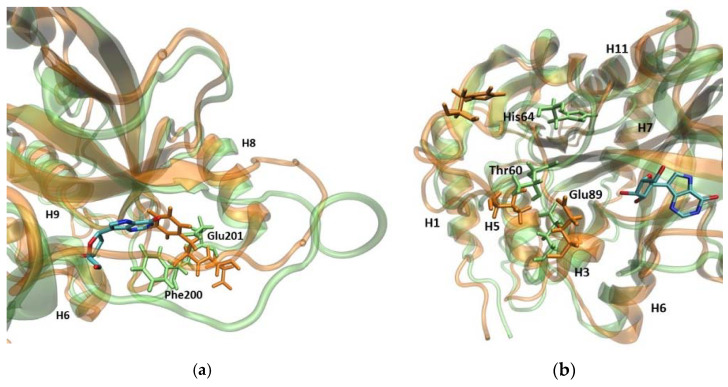
Superposition of the trajectory snapshots showing the active site amino acids in the WT1 PNP (orange) and WT3 PNP (green). The substrate analogue (9-deazainosine) bound to the biologically active WT3 PNP is also shown (colored by the atom types) to better visualize the changes in the geometry of the active site pocket occurring in the WT1 PNP. The ligand was not present in the simulations, its location is from the X-ray structure of the WT PNP trimer (PDB 1A9P [21]). (**a**) In the monomeric enzyme, Phe200 is solvent-exposed, while in the trimeric form, it is located at the monomer–monomer interface, so when neighbouring subunits are not present, Phe200 moves into a hydrophobic pocket within the monomer. This causes Glu201 to shift away from the active site pocket of the enzyme. (**b**) When the loop containing the active site His64 changes its position in the WT1 PNP, the interaction between Thr60 and Glu89 is broken, causing Glu89 to also displace.

As previously indicated by the RoG values, the active site of PNP loses its original shape, as observed in the WT1 PNP simulation. Although the largest contribution to this conformational change comes from the movement of the loop 59–66, on which His64 is placed, the pronounced differences (in both replicates during the last 50 ns of the simulation) of positions observed for residues Glu89 and Glu201 also occur (Figure 5).

In the WT1 PNP, because of the hydrophobic effect, Phe200 moves towards the active site center. This movement prevents the Glu201 side chain from positioning inside the active site and forming the correct spatial “pocket” for the substrate, as is the case for the WT3 PNP trimeric enzyme. Due to the Phe200 steric disturbance, Glu201 is directed away, causing this residue to protrude, instead of forming the proper active site conformation, i.e., the conformation enabling the hydrogen bond with the substrate purine base (Figure 5a).

Another important change regards Glu89, which, in the WT3 PNP, forms a stable interaction with Thr60. By contrast, in the WT1 PNP, when the loop containing His64 moves away from the active site, the interactions of Glu89 with Thr60 are lost, causing a change in the position of the Glu89 side chain (Figure 5b).

Such changes must have significant consequences for the enzymatic properties of the WT1 PNP, as according to the accepted molecular mechanism of trimeric PNPs, the hydrogen bond between Glu201 and the N1 position of the substrate purine base is a must for catalysis, while Glu89 is proposed to form a catalytic triad with His86 and phosphate [12].

MD simulations strongly suggest that the monomeric form of the calf PNP, due to the rearrangement of amino acids building the active site, may not have the catalytic activity displayed by the natural trimeric enzyme. We further decided to check this hypothesis experimentally. The next step was, therefore, the attempt to obtain the calf PNP in a monomeric form and study its properties in vitro. For this purpose, we specifically looked for residues that, when mutated, would destabilize interactions at the interface of the subunits and, ultimately, lead to the formation of a monomeric form of the protein.

#### 2.1.2. Selection of Interface Amino Acids for Mutation

The mutation sites that were chosen, based on analyzing the hot spots and alanine scanning of the subunit–subunit interface, are presented in Table 1. Hot spot analyses pointed to amino acids stabilizing the subunit interface, and alanine scanning quantified which residues contribute most to keeping PNP in a trimeric form. The six selected amino acids, namely Met87, Ser142, Arg148, Phe159, Asn199, and Glu205, located at the interface of the subunits, were substituted with alanines.

**Figure 6:**
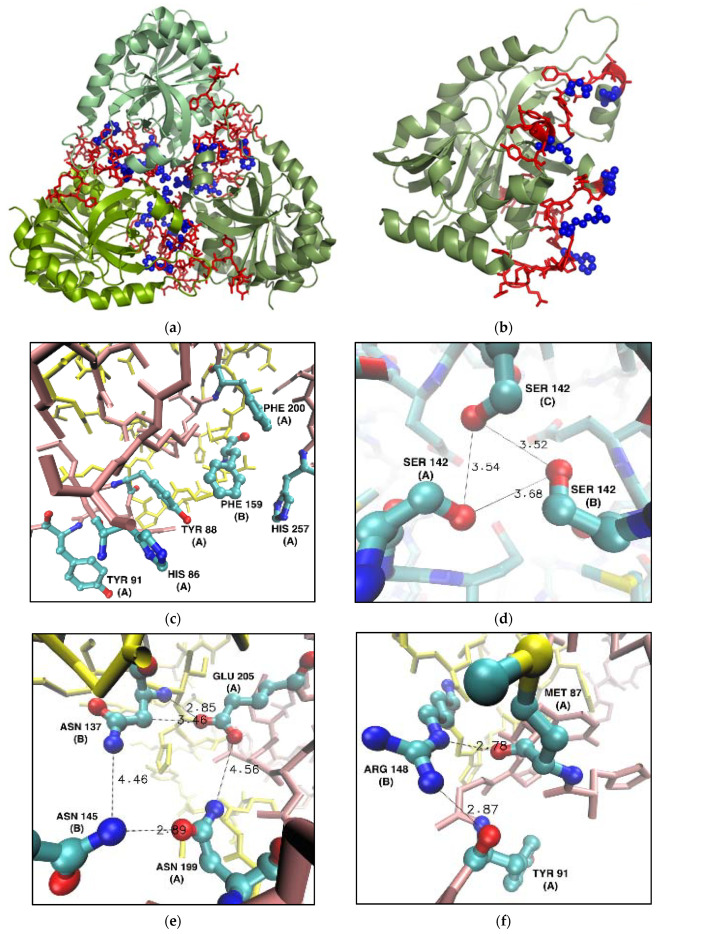
Residues selected for mutation into alanine in the WT calf PNP (PDB ID 3FUC [27]). Non-mutated amino acids belonging to the monomer–monomer interface (red) and six mutated (blue) residues localized on the interface shown in the overall structure of the calf PNP trimer (**a**) and one monomer (**b**). (**c**–**f**): Fragments of the monomer–monomer interface (here shown for monomers denoted A and B, chain A—pink, chain B—yellow) focusing on the locations of mutated residues. Chain names are given in parentheses. Distances are in Å. For clarity, only the heavy atom positions are shown. (**c**) Phe159 from the cluster of six aromatic residues; (**d**) Ser142 forming a hydrogen bond network in the center of the trimer; (**e**) Asn199 and Glu205 forming the hydrophilic cluster with Asn137 and Asn145 form the neighbouring subunit; (**f**) Arg148 and Met87 were mutated because Arg148 hydrogen bonds with Tyr91 and Met 87 from the neighbouring subunit.

Figure 6 shows the locations of the residues selected for mutations in the overall structure of the trimeric PNP and the monomeric subunit (panels a, b), with insets of the interface zooming on these residues (Figure 6c–f). Hydrogen bonds and hydrophobic interactions, crucial to the formation of the interface between subunits, are also shown.

Figure 6c shows a cluster of six aromatic residues forming the PNP ligand binding site and interacting via stacking. However, the central Phe159 is not in direct contact with PNP substrates and is the only residue of the active site belonging to the neighbouring subunit. As mentioned in the Introduction, the mutation of Phe159 into alanine does not significantly change the enzymatic activity, when compared to the activity of the WT PNP [16]. However, Table 1 shows that replacing Phe159 with alanine would exert a destabilizing effect on the monomer–monomer interface of about 2–3 kcal/mol, so we picked this residue for mutation.

Asn137 and Asn145 of one subunit form, with Asn199 and Glu205 of the other subunit, a hydrophilic cluster on the monomer–monomer interface (Figure 6e). Replacing just two of the four residues with alanine should significantly disrupt this connection. Based on the data from the hot spot servers and alanine scanning, we selected Asn199 and Glu205 for their replacement with alanine.

Arg148 of one subunit forms two hydrogen bonds with Met87 and Tyr91 of another subunit (Figure 6f). Therefore, replacing Arg148 and Met87 with alanines should disrupt these favorable interactions on the monomer–monomer interface, as confirmed by in silico alanine scanning (Table 1).

Finally, Ser142 shapes up the specific contact point between three subunits (Figure 6d). Each serine interacts with one another, forming a hydrogen bond network between the hydroxyl groups in the middle of the trimeric enzyme (Figure 6d). Even though Ser142 did not show up in the analysis of the interface hot spots, we decided to mutate it because of the possible destabilizing effect of its deletion on the tertiary structure of the trimer.

### 2.2. In Silico Studies of the Mutated PNP Forms (6Ala1 PNP and 6Ala3 PNP)

The effects of the six selected mutations on the overall stability of the PNP molecule were first investigated with the aid of 150 ns long molecular dynamics simulations conducted in two replicas. The simulations were performed for two forms of the mutated protein, the trimer (6Ala3 PNP) and the monomer (6Ala1 PNP). These simulations allowed us to assess whether it was worth obtaining the 6Ala mutant in vitro.

#### 2.2.1. 6Ala1 PNP vs. WT3 PNP and WT1 PNP

As expected, the trajectory-derived conformations of the PNP monomers deviate from their starting structures more than the WT3 PNP ones (as shown in Figure 2). In the last 50 ns of the simulation, the average RMSD for both monomeric forms, mutated, 6Ala1 PNP (3.34 ± 0.2 Å), and with the wild-type sequence, WT1 PNP (2.84 ± 0.1 Å), is about 1 Å higher than the RMSD for the trimeric WT3 PNP (1.94 ± 0.1 Å). Such dynamical behavior propagates to the active site, as also highlighted in the different radius of gyration (RoG) values, which is a measure of the compactness of the structure.

The average RoG of the active site residues calculated for the last 50 ns of the 6Ala1 PNP mutant simulation (10.3 ± 0.2 Å) is larger by about 0.8 Å than the one of the WT3 PNP (9.6 ± 0.1 Å), but it is similar to the WT1 PNP one (10.4 ± 0.2 Å), (Figure 2b). However, if His64 in the flexible loop is excluded from the calculation, the differences are much smaller, as RoG is 9.7 ± 0.2 for 6Ala1, 9.2 ± 0.1 for WT1, and 9.4 ± 0.1 for WT3 PNP forms (Figure 2c).

**Figure 7:**
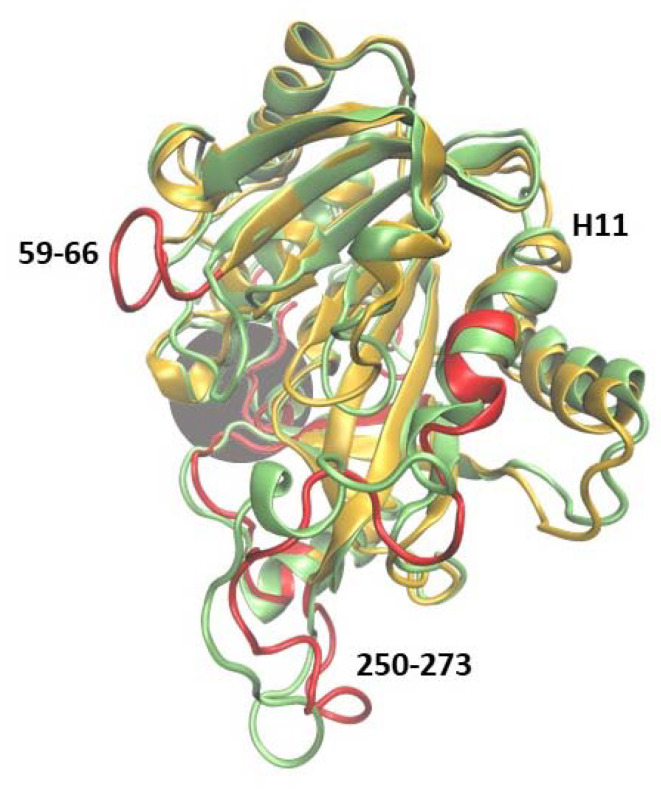
Superposition of two trajectory-derived structures, 6Ala1 PNP (yellow, captured at 150 ns) and one subunit of the WT3 PNP (green, at 150 ns). The gray area marks the approximate location of the active site. The largest displacements observed in the 6Ala1 PNP are marked red and, in principle, are similar to those observed in the case of WT1 PNP (compare with Figure 4), but in the mutant, additionally, the region 250–273 exhibits more flexibility than in the WT1 PNP and WT3 PNP.

The main structural changes between 6Ala1 PNP and WT3 PNP are depicted in Figure 7. They include the same regions as for the wild-type monomer (see Figure 4). However, in the mutant, additionally, the region 250–273 exhibits more flexibility than in the WT1 PNP and WT3 PNP. This region contains a part of helix H11 and is solvent-exposed in the trimeric form of the protein.

**Figure 8:**
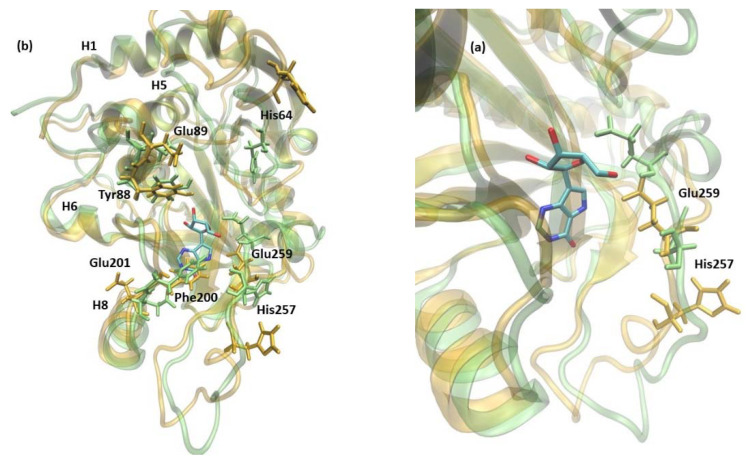
The shift of active site amino acids observed in the structure of 6Ala1 PNP (yellow), in comparison with the WT3 PNP (green). The substrate analogue (9-deazainosine) bound to the biologically active WT3 PNP is also shown (colored by the atom types) to better visualize the changes in the geometry of the active site pocket occurring in the 6Ala1 PNP. The ligand was not present in the simulations, and its location is from the X-ray structure of the WT PNP trimer (PDB 1A9P [21]). Changes are similar to those already observed for the WT1 PNP and include residues His64, Tyr88, Glu89, Phe200, and Glu201 (**a**) (compare with Figure 5). In the 6Ala1 PNP monomer, His257 additionally moves away from the active site, as the adjacent helix partially unwinds. Glu259 also changes its position in the 6Ala1 mutant, as shown on the focus to this area, depicted in panel (**b**).

Additionally, in the 6Ala1 PNP, the active site changes its shape (Figure 8), as already observed for the WT1 PNP (compare with Figure 5). Here, also, the residues His64, Tyr88, Glu89, Phe200, and Glu201 change their positions—the loop on which His64 is placed moves away from the active site pocket, Glu201 is directed away from the active site because of the burying of Phe200, and the helix on which residues 88–89 are placed fluctuates a lot. Moreover, in the 6Ala1 mutant, an increase of the RMSF of His257 and (in one replicate) Glu259 are observed, as well. The His257 of the 6Ala1 PNP is moved outside of the active site, as the helix on which it is placed unwinds in one of the replicates of the 6Ala1 simulations. However, in another replicate, the helix on which His257 and Glu259 are placed moves away from the binding site in its entirety, likely as a result of the overall destabilization of the structure.

Overall, this destabilization of the active site observed in the simulations of the 6Ala1 PNP again, as in the case of WT1 PNP, suggests that the monomeric form of the protein is incapable of catalysis.

#### 2.2.2. 6Ala3 PNP vs. WT3 PNP

Because the mutated monomer displayed different locations of the active site residues, we further checked in silico if incorporating the same mutations to the PNP trimer would influence the conformational stability of such an assembly. Therefore, the molecular dynamics simulations of the trimeric PNP with six mutations (6Ala3 PNP) were executed.

Introducing mutations to the WT trimer did not drastically change the RMSD, such as in the case of both monomeric enzyme forms. As depicted in Figure 2a, the RMSD values plotted as a function of the simulation time, for both structures WT3 PNP (black line) and 6Ala3 PNP (blue), stabilize over the last 50 ns of the simulation at the level of about 2 Å.

**Figure 9:**
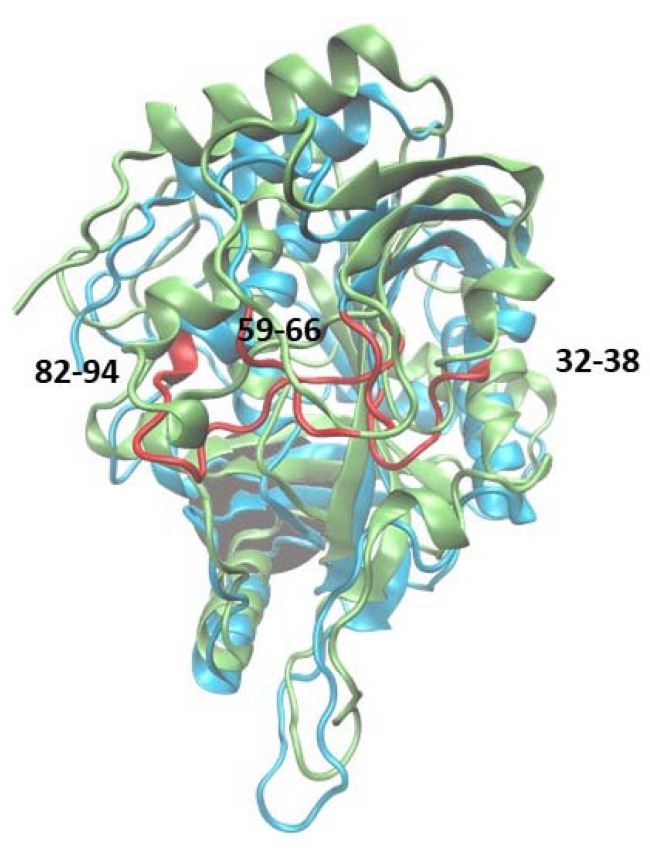
Superposition of the WT3 PNP (one subunit) and 6Ala3 PNP structures observed after 150 ns of MD simulations. The structure of the 6Ala3 PNP is in blue, and WT3 PNP is in green. The regions 32–38, 59–66, and 82–94, which show the largest displacements in the 6Ala3 PNP, as compared to WT3 PNP, are shown in red.

Additionally, fluctuations of the 6Ala3 PNP are much smaller than those observed for the WT1 and 6Ala1 PNP forms. However, in the mutated trimer, an increase in fluctuability of certain parts of the protein is observed, namely in the regions 32–38, 59–66, and 82–94 (Figure 9). These regions also exhibit larger fluctuations in the 6Ala1 mutant; however, some of the regions characterized by higher fluctuations for the 6Ala1 PNP are stabilized in the structure of the 6Ala3 enzyme form.

The 6Ala3 PNP mutant also demonstrates some changes in the conformation of the active site; however, the average RoG of the active site residues (9.5 ± 0.1 Å) is fairly similar to that observed for the WT3 PNP (9.6 ± 0.1 Å). The loop on which His64 is placed does not move away from the active site completely, as in the simulations of both monomers. It shifts somewhat away from the active site, resulting in slightly higher fluctuations of the His64 residue (Figure 3). The change in the position of Glu89 is also observed. However, in this case, it can be attributed to the Arg148Ala mutation. Namely, the space usually occupied by the large arginine side chain from a neighbouring monomer is freed up in the 6Ala3 mutant, causing the loop on which Tyr88 and Glu89 are placed to move away from the active site.

These results indicate that, unlike the biologically existing WT3 PNP, the 6Ala3 mutant in the trimeric form may not have catalytic ability, due to the lack of proper interactions between subunits, stabilizing not only the trimeric structure, but also the correct conformation of amino acids building the active site.

### 2.3. In Vitro Studies of the 6Ala PNP

MD simulations show that the WT1, 6Ala1, and 6Ala3 PNPs structural dynamics of the monomers are overall similar to that observed for the biologically active WT3 PNP. However, in the monomeric enzyme forms, crucial changes occur in two (or three) helical parts of the protein, in some loops and, most importantly, in the positions of amino acids building the active site. These changes probably hamper the binding of substrates, and even if binding occurs interactions of the purine and nucleoside substrates with the catalytic Glu201 are not possible. This, in turn, is expected to lower, or even diminish, enzymatic activity. To check this hypothesis, we prepared the 6Ala mutated form of the enzyme (6Ala PNP) in vitro and experimentally studied its structural properties and enzymatic activity. The 6Ala PNP was obtained as described in the Materials and Methods section. Its tertiary and secondary structures were characterized by analytical ultracentrifugation and CD spectral analysis, respectively.

#### 2.3.1. Oligomeric State of 6Ala PNP Deducted from Analytical Ultracentrifugation

**Figure 10:**
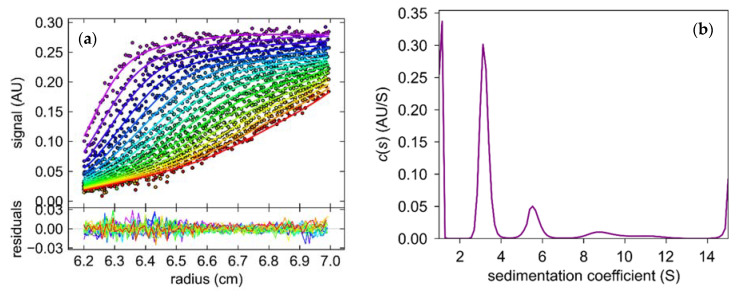
The results of the sedimentation velocity experiment with absorption detection obtained for the mutant 6Ala PNP at 42,000 rpm, 20 °C, 0.6 M Arginine pH 7.0 (**a**) The concentration distributions as a function of radial distance (r). The curves from purple to red illustrate the shifting of the sedimentation boundary during the experiment. (**b**) Sedimentation coefficient (S) distribution.

The sedimentation coefficient (S) obtained from the sedimentation velocity experiments revealed that samples of 6Ala PNP contained several forms of the mutant protein (Figure 10). One form, with S equal 2.9–3.2 S, was more abundant, as it constituted 50–60% of the total protein in the sample, whereas the second form, with S equal 5.8 S, was less populated and constituted only about 10% of the total protein. Small fractions with higher sedimentation coefficients were also present, with 8.7 S—10% and 11 S—5% of the sample. We calculated the theoretical sedimentation coefficient S for the monomeric and trimeric forms of the calf PNP. For the monomer, the S value extrapolated from the crystallographic structure of the trimeric PNP using the program HYDROPRO was equal to 2.9 S, while for the trimer, it was calculated as equal to 5.8 S. Hence, the analytical ultracentrifugation experiment showed that most of the protein exists in the monomeric form, about 10% forms a trimer, and higher oligomers are also observed.

#### 2.3.2. Secondary Structure Analysis from CD Spectra

**Figure 11:**
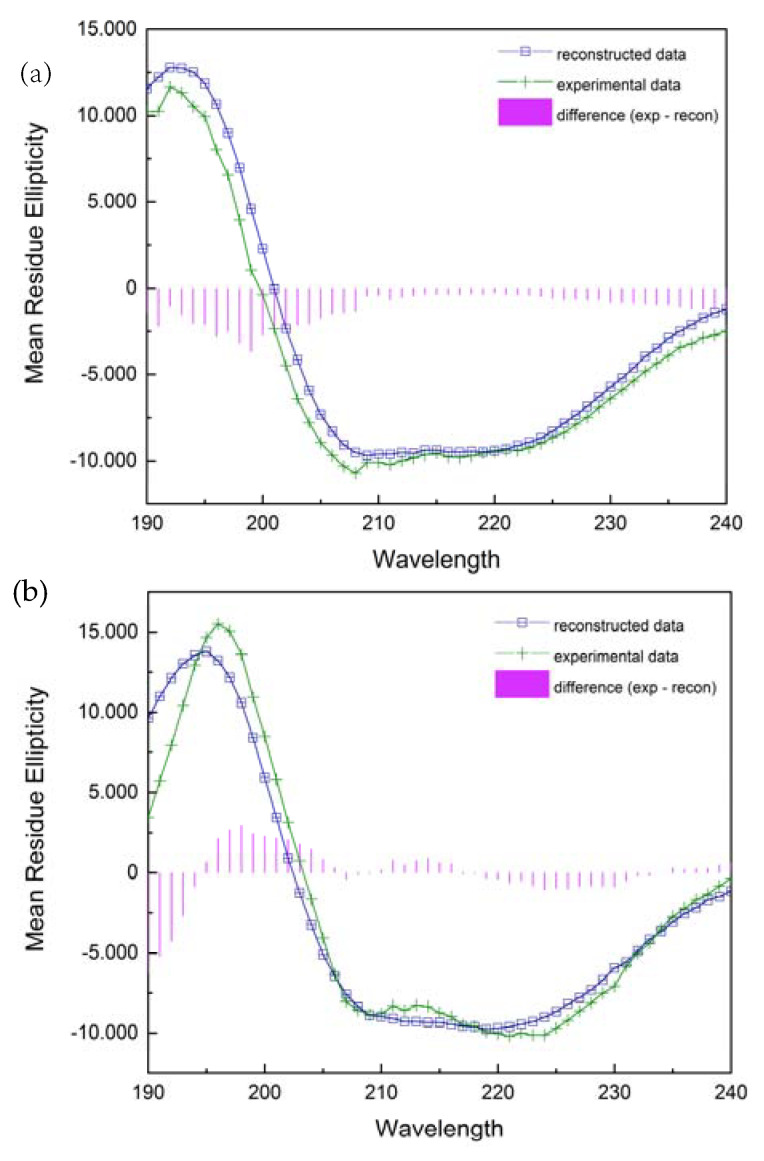
Experimentally collected CD spectra (green) of the 6Ala PNP (**a**) and the WT PNP (**b**) and their reconstruction with the DichroWeb/Selcon 3 program (blue).

The far ultraviolet circular dichroism spectra of two various forms of the PNP protein are shown in Figure 11. A broad negative band extending from 205 to 225 nm and a positive band with a maximum at 195 nm for the wild-type PNP and at 192 nm for 6Ala1 PNP indicate a significant contribution to the spectrum of the β-strands and alpha-helices. Although the spectra exhibit some differences, their evaluation by DichroWeb Program/Selcon 3 [28] suggests that there are only minimal differences in the secondary structure of the 6Ala mutant, when compared to the structure of the WT PNP. Especially, as depicted in Table 2, the secondary structure content of the 6Ala PNP mutant is nearly identical to that obtained for the X-ray apo form of WT PNP (PDB ID 1PBN, 21). However, such analysis of the CD spectra must be interpreted with caution, as there is discussion in the literature that the content of structures determined by the CD method differs from the results obtained by X-ray diffraction. Nevertheless, in the case of 6Ala1 PNP, the results allow to conclude that the secondary structure of the mutant is compatible with the WT structure obtained crystallographically.

#### 2.3.3. Enzymatic Activity Measurements

**Figure 12:**
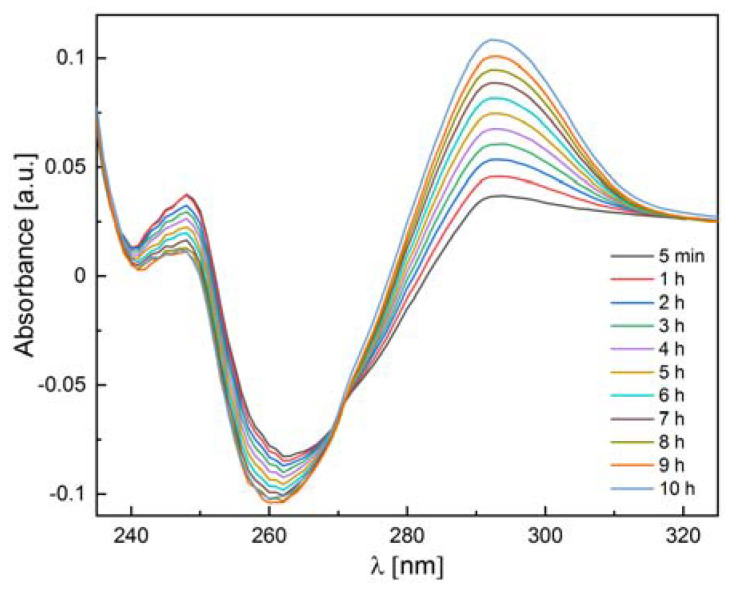
Differential absorbance spectra for the 6Ala PNP mutant PNP (0.11 mg/mL) incubated with 200 μM inosine in 50 mM phosphate buffer pH 7.0 at 25 °C. Spectra (in 1 cm path length cuvette) were measured after 5 min from the start of the reaction (black line) and, next, every hour.

We also checked whether the 6Ala PNP mutant exhibits enzymatic activity toward natural substrates of the WT PNP, i.e., inosine and 7-methylguanosine. Since calf PNP does not catalyze adenosine phosphorolysis [12], this nucleoside was used as a negative control. Our measurements confirmed the phosphorolytic activity of the WT PNP for inosine and 7-methylguanosine and the lack of such activity for adenosine. By contrast, as depicted in Table 3, the 6Ala PNP mutant showed extremely low phosphorolytic activity for all three substrates: inosine (see also Figure 12), 7-methylguanosine, and adenosine. Therefore, it is justified to speculate that the mutant has no enzymatic activity at all and that the observed residual activity is the result of some *E. coli* PNP traces present in the samples, which come from the expression of the mutated enzyme in the host *E. coli* cells.

## 3. Discussion

Almost half (49%) [3] of the unique amino acid sequences of enzyme structures deposited in PDB form oligomers. This ratio shows that proteins, including enzymes, must profit somehow from oligomerization, although these profits may be quite different. Subunit cooperation in ligand binding (and catalysis) is the first benefit that comes to everyone’s mind when thinking about oligomeric proteins, with hemoglobin at the forefront, which is a classic textbook example of positive cooperativity [29]. The most famous enzyme exhibiting negative cooperativity is probably D-glyceraldehyde-3-phosphate dehydrogenase (GAPDH). Although several groups of scientists were kept busy for many years unveiling its enzymatic properties, the molecular basis of strong negative cooperativity resulting in the so-called half-of-the-sites binding [3] and its role in catalysis still hides some secrets [30]. Regulating protein activity through the ability to dissociate or associate subunits, depending on the concentration of the protein or the presence of its ligands, also appears to be an evident potential benefit of homooligomerization. For example, adenylosuccinate synthetase, which operates at a branch point of the de novo synthesis of purine nucleotides and the purine nucleotide cycle, the so-called salvage pathway, is not active as a monomer, but is activated by a substrate-induced dimerization [31]. None of these benefits, however, apply to the trimeric PNP. Although dissociation into monomers with higher activity than the trimer [32] and the negative cooperation in the binding of transition state analogues [5] were proposed some time ago for this enzyme, subsequent studies have led to the rejection of these hypotheses. It was proven that the trimer is very stable, constituting a so-called obligatory oligomer [14,20], and the subunits forming this assembly function independently, in the ground and transition states [7]. Therefore, for PNP the benefits from the trimeric architecture must have other reasons.

Molecular dynamics simulations of the wild-type trimeric calf PNP and the individual monomer “extracted” in silico from the trimer revealed marked differences in the dynamical behaviour of these two protein forms. The overall shape of both variants differs only slightly, but some parts, including the substrate binding site, are significantly affected when no subunit neighbours are present. As a result, in the monomeric molecule, the active site loses its original shape. Namely, Phe200 moves into the active-site pocket, not only occupying the space devoted to the nucleoside substrate, but also directing Glu201, which is crucial for catalysis, not into, but outside of the active site. Moreover, Glu89 proposed to form a catalytic triad with His86, and the phosphate markedly changes its position. This indicates that one individual subunit, although it has all the residues necessary to build the active site, probably cannot preserve the proper architecture of the active site pocket necessary to conduct catalysis.

To verify this hypothesis provided by the MD simulations, we designed a mutant, 6Ala PNP, in which six residues, predicted by us as crucial for the trimer formation, were replaced with alanines to disturb the interface of subunits. The MD simulations showed that, in this mutant, despite only small changes in the overall structure, the active site of the monomeric form, 6Ala1 PNP, also did not hold onto its original shape, suitable for catalysis, and, similar to the case of the non-mutated monomer obtained in silico (WT1 PNP), the catalytic Glu201 was directed away from the nucleoside substrate making this PNP form unable to perform efficient catalysis, as further verified by us experimentally. In the trimeric form of the 6Ala PNP mutant, changes in the active site are much less pronounced. However, fluctuations of some of the key active site residues can also be observed in the case of this enzyme form, especially of His64 and the loop on which His64 is located. This suggests that, in the case of 6Ala PNP, due to weaker contacts between monomers, the trimeric form does not have catalytic activity.

With such unequivocal in silico results indicating that the monomeric form of PNP could not exhibit the catalytic activity, we decided to verify these conclusions by in vitro experiments. The 6Ala mutated form of the enzyme (6Ala PNP) was prepared, and its secondary and tertiary structures, as well as its catalytic properties, were characterized.

In analytical ultracentrifugation, both forms, the monomer and trimer, were detected, as well as a small fraction of larger assemblies. However, the dominant form of the mutant 6Ala PNP present in the solution was a monomer (50–60%). The observed small amounts of trimeric forms (10%) can be explained by the fact that, from the several amino acids on the surface between the monomers forming the biologically observed trimer, only six were mutated to alanine. The other non-mutated residues could still interact with one another, resulting in the formation of small amounts of trimeric forms. Additionally, random combinations of monomers or trimers could explain the detection of higher order oligomers.

The overall secondary structure of the 6Ala1 PNP mutant, from the CD measurements, is consistent with the crystallographic data for the WT PNP. Additionally, the structure of the mutant contains 2.4% more turns and unordered structures, 3% fewer ⍺-helices, and 1% fewer β-sheets than the WT PNP.

The observed minimal catalytic activity towards inosine and 7-methylguanosine as substrates (10^−5^–10^−6^ lower than that of the WT calf PNP) indicates that this activity could come from the trimer. However, the observed activity for adenosine, which is not the substrate of the calf enzyme, but only of the hexameric PNPs, contradicts this hypothesis. Since the active site was not modified and MD simulations showed that the arrangement of the active site amino acids would likely result in the loss of activity, it rather appears that the *E. coli* PNP present during the overexpression of 6Ala PNP in the host cells is responsible for the apparent residual activity. We have already paid attention to this phenomenon before [33].

Despite the lack of sequence homology, the mammalian and *E. coli* PNP show similar secondary and tertiary structures of the subunits [12]. For the *E. coli* PNP, we have previously shown that oligomerization is necessary for its stability and, thus, for the catalytic activity of this hexameric enzyme [8]. This work indicates that this is also the case for the trimeric mammalian PNP. In both enzymes, the adjacent subunits form a scaffold for each other, which stiffens the structure of the whole molecule, including, in particular, the stabilization of the active site pocket. This phenomenon can, in a sense, be regarded as similar to allosteric regulation, since changes in one certain part of the subunit, namely at the subunit–subunit interface, affect the structure of the active site.

## 4. Materials and Methods

### 4.1. Materials

Wild-type PNP was obtained and purified as previously described [34]. Adenosine, inosine, and xanthine oxidase from bovine milk (1 U/mg) were obtained from Sigma-Aldrich (St. Louis, MO, USA). The 7-methylguanosine was synthesized from guanosine according to Jones’ and Robins’ method involving methyl iodide [35]. This approach assured the preparation was free from sulphate, which as an ion-resembling phosphate could bias the results. All other chemicals were purchased from Roth (Karlsruhe, Germany) or Sigma-Aldrich and were of the highest available purity.

### 4.2. Molecular Modeling—System Preparation

The crystal structure of the apo form of PNP from the calf spleen (PDB code: 1PBN) [21] was used as an initial structure for the monomer and trimer enzyme models. Polar hydrogen atoms were added using the WHAT IF software [36], while non-polar atoms were added using Amber 12 [37]. The protein atoms were parametrized using the Amber ff99SB force field [38]. Na^+^ ions were added to neutralize the system [39]. Using the procedure described above, four systems were prepared for MD simulations: WT3 PNP and its mutant with disrupted interactions between subunits (6Ala3 mutant), as well as their monomeric variants (WT1 PNP and 6Ala1 mutant).

The trimeric structure of the PNP apoenzyme after 500 ps of molecular dynamics simulations was used as a template to introduce 6 mutations to obtain trimeric PNP (6Ala3 PNP) and two monomeric forms (WT1 PNP and 6Ala1 mutant). The Maestro 9.1 software was used to introduce in silico mutations [40].

### 4.3. Molecular Dynamics Simulations

All systems were energy minimized in a 5-step procedure (described in detail in [8]). After energy minimization (geometry optimisation), systems were equilibrated for 500 ps. During the first 300 ps of equilibration, the NTV ensemble was used. The temperature was linearly increased from 0 K to 300 K during the first 250 ps and kept constant at 300 K during the preceding phases of simulations. The second phase of equilibration (300–500 ps) was at constant temperature (300 K) and pressure (1 atm) using the NTP ensemble. Following the geometry optimization and equilibration, we performed 150 ns molecular dynamics (MD) simulations using Amber 9 [41]. The production phase (10 ns) was performed in the NTP ensemble at a constant temperature of 300 K and 1 atm pressure.

Each simulation was conducted in duplicate, and both replicas were examined. Trajectories were analyzed using Gromacs [42] and Amber tools and visualized with VMD [43] and UCSF Chimera [44]. Root mean square fluctuations (RMSF) were calculated for heavy atoms and root mean square deviation (RMSD) for the C_⍺_ alpha atoms, with respect to the starting structure. The radius of gyration (RoG) and RMSF of the active site were calculated, taking into account the following residues: Ser33, His64, Arg84, His86, Tyr88, Glu89, Ala116, Ala117, Gly118, Gly119, Tyr192, Phe200, Glu201, 217, 218, Met219, Ser220, Thr242, Asn243, Lys244, Vel245, His257, and Glu259. Phe159 was not included, since it is not present in the monomeric form because it belongs to the neighbouring subunit. Additionally, the radius of gyration was also calculated using all of the aforementioned residues, but excluding His64.

### 4.4. Prediction of the Mutation Sites Leading to the Monomeric Enzyme Form

Two approaches based on empirical scoring functions were used to predict the optimal sites for mutations that would disrupt the subunit–subunit interface and result in a monomeric form of the calf PNP in vitro. The analysis of hot spots that indicated the energetically crucial amino acids at the interface was performed employing the following servers: PISA, KFC, and HOT POINT [15,22,23]. Alanine scanning to estimate the change in the binding free energy (ΔΔG) of the subunit–subunit interface upon amino acid mutation to alanine was performed with the CUPSAT, I-MUTANT 2.0, and ROBETTA BETA servers [24,25,26].

Based on these analyses, six residues were chosen for mutation into alanine: Met87, Ser142, Arg148; Phe159, Asp199, and Glu205 (Table 1).

### 4.5. Site-Directed Mutagenesis

To obtain the monomeric mutant of PNP, site-directed mutagenesis was performed according to the manufacturer’s instructions, using Quick Change Lightning Multi Site-Directed Mutagenesis Kit (Stratagene, La Jolla, CA, USA). The primers chosen for amino acid replacement are collected in Table 4. The substitution of the desired amino acid was achieved in the sequence coding for the WT PNP inserted with the pET28a+ plasmid [34], using PCR reactions. The mutations were confirmed by sequencing.

### 4.6. Expression Optimization and Obtaining the 6Ala PNP Mutant

One colony of *E. coli* BL21(DE3) strain carrying the pET28a(+): PNP with the selected mutations was used to inoculate 5 mL of the LB broth containing kanamycin (30 μg/mL) and grown overnight at 37 °C. The next day, 2 mL of the culture was added to 100 mL of the medium with kanamycin and incubated at 37 °C, with shaking to OD_600_ = 0.7. The new culture was then induced with 0.5 mM of IPTG and grown for 3 h at the same conditions. The cells were then harvested, resuspended in 50 mM Tris-HCl, pH 7.6, and 0.3M NaCl, and frozen at −20 °C. Cell lysis was achieved by the repeated freezing/thawing procedure and, finally, cell sonication (4 pulses, 30 s each). The obtained solution was then digested by DNAse, RNAse at 4 °C for 15 min, and centrifuged at 8000× *g* for 15 min. The pellet was next washed twice with a 0.2% Triton X-100 and collected. The pellet, which contained 6Ala PNP mutant in the inclusion bodies, was dissolved in 6M guanidinium hydrochloride. The suspension was stirred for 12 h at 4 °C and centrifuged. Folding was achieved, according to [45], in 0.6 M arginine and pH 7.0 because folding in a typical buffer, e.g., Tris pH 7.0, resulted in substantial protein aggregation. Therefore, to prevent excessive aggregation, the arginine buffer was added to the stirred protein in guanidinium hydrochloride (60× times dissolved guanidinium hydrochloride), and stirring was continued for 15 min, followed by gentle shaking for the next 30 min. The protein was then concentrated on the Millipore filter.

### 4.7. Circular Dichroism Spectra

CD spectra measurements were performed on Bio-Logic SAS (SFM-400/QS, MOS-450/AF-CD) and Chirascan spectrophotometers. The measurement was carried out in 10 mM phosphate buffer with a protein concentration of about 0.3 mg/mL at 25 °C, using 1 mm and 0.1 mm quartz cuvettes. Spectra in the range 188–250 nm were collected at a scan speed of 20 nm per minute. Eight scans were averaged for each spectrum.

Data were analyzed on the DichroWeb using the SELCON3 program [28] to calculate the secondary structure elements of both enzyme forms.

### 4.8. Activity Measurements

Enzyme activity measurements were performed on a double-beam spectrophotometer (Cary 100; Agilent, Santa Clara, CA, USA) equipped with a Peltier thermostat for both the experimental and reference cuvette holders, according to the methods described earlier [8], in 50 mM phosphate buffer pH 7.0 at 25 °C, using a direct spectrophotometric assay for adenosine and 7-methylguanosine as substrates, as well as a coupled assay with xanthine oxidase in the case of inosine [46,47,48]. Initial substrate concentrations were in the range 100–200 μM, and PNP mutant concentrations 0.06–0.16 mg/mL. Since for the 6Ala PNP mutant, extremely low activity was expected, the method developed previously for dimeric mutants of the hexameric *E. coli* PNP was used [8]. Namely, the progress of the reaction was followed by differential absorption spectra collected in the range 230–320 nm every 5 min, or even less frequently, as needed. Initial and final spectra for the experimental and reference cuvettes vs. buffer were also measured to determine if the overall changes in the spectra in the experimental cuvette corresponded to the phosphorolytic reaction and to control the stability of the substrates in the absence of the mutant (reference cuvette).

### 4.9. Analytical Ultracentrifugation

The sedimentation velocity experiment was performed with an analytical centrifuge (Optima XL-I, Beckman-Coulter Inc., Indianapolis, IN, USA) equipped with the absorbance detection system with An-50Ti and An-60Ti analytical rotors and standard double sector Epon-charcoal centerpieces 1.2 cm cells with sapphire windows. Sample concentration was 0.3 mg/mL (390 μL) in 0.6 M Arginine pH 7.0, obtained by HCl titration. After temperature equilibration at 20 °C, the rotor was accelerated to 142,000 g (42,000 rpm), as measured at the bottom of the cell. Data were acquired every 5 or 7 min. The partial specific volume of the mutant, density, and viscosity of the buffer were calculated using the SENTERP program [49,50]. Sedimentation coefficient was calculated with HYDROPRO [51] using the structure of PNP from PDB, entry ID 3FUC [27]. This program computes the basic hydrodynamic properties, i.e., the sedimentation coefficient from the coordinates of the molecule. To obtain sedimentation coefficient and standard sedimentation coefficients s20 from the sedimentation velocity profiles, the Sedfit program with the continuous sedimentation coefficient distribution c(s) model [52] was used.

## Figures and Tables

**Table 1:** The results of the hot spot analysis (the residues selected as hot spots by the servers are marked with the “plus” sign) and free energy change predicted by the alanine scanning approach (the “minus” sign denotes a destabilizing effect on the monomer–monomer interface upon mutation).

				ΔΔG (kcal/mol)		
	PISA [15]	KFC [22]	HotPoint [23]	CUPSAT [24]	I-MUTANT 2.0 [25]	ROBETTA BETA [26]
Met87	+		+	-3.59	−1.53	−0.64
Ser142				−2.51	−0.50	−1.80
Arg148	+	+		−0.98	−0.73	−2.10
Phe159		+		−3.50	−1.73	−2.70
Asn199	+	+	+	−5.15	−1.4	−5.18
Glu205	+	+	+	−0.27	−1.21	−1.5

**Table 2:** Secondary structure elements in the mutant 6Ala PNP and WT PNP, according to the analysis of their CD spectra in DichroWeb.

	α-Helices [%]	β-Strands [%]	Turns [%]	Unordered [%]
WT PNP estimated from the X-ray structure *	30.4	22.8	46.7	
WT PNP CD	31	22	20.5	25.4
6Ala PNP CD	28	21	22.9	27.9

* Calculated for the structure 1PBN in The Secondary Structure Server (https://2struc.cryst.bbk.ac.uk/twostruc, accessed on 20 September 2022).

**Table 3:** Comparison of the phosphorolytic activity of the 6Ala PNP mutant obtained during prolonged scanning kinetic experiment, and the WT PNP activity; 50 mM phosphate buffer, pH 7.0, 25 °C.

Substrate	6Ala PNP (U·mg^−1^)	WT PNP (U·mg^−1^)
7-Methylguanosine	4.2 × 10^−4^	102
Inosine	1.4 × 10^−4^	34
Adenosine	7.4 × 10^−4^	-

**Table 4:** Primer sequences used in the PCR reaction.

Mutation Sites	Primer Sequence
Met87	5′ cgg ata gcc ttc ata cgc gtg gaa cct gcc ctg 3′
Ser142, Arg148	5′ ctc att ggg ccc tgc gag agg gtt ctc acc agc gaa acc agg tag 3’
Phe159	5′c aga cat ggc agg ggc acg aac tcc aaa cct ttc 3
Asn199, Glu205	5′ ag cag gcg aca cgc tgc cac agt ctc aaa agc ggg acc ccc caa c 3′

## Data Availability

Data and materials will be made available upon reasonable request (please send to AB or BWK).

## Abbreviations

PNP: purine nucleoside phosphorylase; WT PNP, wild-type enzyme; 6Ala PNP, mutant with six amino acids at the subunit–subunit interface (Met87, Ser142, Arg148, Phe159, Asp199, and Glu205) changed to alanine residues; WT3 PNP, trimeric form of the wild-type calf PNP; WT1 PNP monomeric form of the wild-type calf PNP (obtained in silico); 6Ala3 and 6Ala1, trimeric and monomeric forms, respectively, of the 6Ala mutant of PNP (obtained in silico); MD, molecular dynamics; CD, circular dichroism; RMSD, root mean square deviation; RMSF, root mean square fluctuation; RoG, radius of gyration.
